# Genome-wide association study and ancestral origins of the slick-hair coat in tropically adapted cattle

**DOI:** 10.3389/fgene.2014.00101

**Published:** 2014-04-29

**Authors:** Heather J. Huson, Eui-Soo Kim, Robert W. Godfrey, Timothy A. Olson, Matthew C. McClure, Chad C. Chase, Rita Rizzi, Ana M. P. O'Brien, Curt P. Van Tassell, José F. Garcia, Tad S. Sonstegard

**Affiliations:** ^1^Department of Animal Science, Cornell UniversityIthaca, NY, USA; ^2^Bovine Functional Genomics Laboratory, United States Department of Agriculture, Agricultural Research ServicesBeltsville, MD, USA; ^3^Department of Animal Science, Iowa State UniversityAmes, IA, USA; ^4^Agricultural Experiment Station, University of the Virgin IslandsSt. Croix, Virgin Islands; ^5^Department of Animal Science, University of FloridaGainsville, FL, USA; ^6^Irish Cattle Breeding FederationCork, Ireland; ^7^Meat Animal Research Center, United States Department of Agriculture, Agricultural Research ServicesClay Center, NE, USA; ^8^Department of Veterinary Medicine, Milan UniversityMilan, Italy; ^9^Division of Livestock Sciences, BOKU University of Natural Resources and Life SciencesVienna, Austria; ^10^Faculdade de Medicina Veterinária de Araçatuba, UNESP - Univ Estadual PaulistaBrazil

**Keywords:** SLICK, Criollo, Senepol, Carora, Romosinuano, thermo-tolerance

## Abstract

The slick hair coat (SLICK) is a dominantly inherited trait typically associated with tropically adapted cattle that are from Criollo descent through Spanish colonization of cattle into the New World. The trait is of interest relative to climate change, due to its association with improved thermo-tolerance and subsequent increased productivity. Previous studies localized the *SLICK* locus to a 4 cM region on chromosome (BTA) 20 and identified signatures of selection in this region derived from Senepol cattle. The current study compares three slick-haired Criollo-derived breeds including Senepol, Carora, and Romosinuano and three additional slick-haired cross-bred lineages to non-slick ancestral breeds. Genome-wide association (GWA), haplotype analysis, signatures of selection, runs of homozygosity (ROH), and identity by state (IBS) calculations were used to identify a 0.8 Mb (37.7–38.5 Mb) consensus region for the *SLICK* locus on BTA20 in which contains *SKP2* and *SPEF2* as possible candidate genes. Three specific haplotype patterns are identified in slick individuals, all with zero frequency in non-slick individuals. Admixture analysis identified common genetic patterns between the three slick breeds at the *SLICK* locus. Principal component analysis (PCA) and admixture results show Senepol and Romosinuano sharing a higher degree of genetic similarity to one another with a much lesser degree of similarity to Carora. Variation in GWA, haplotype analysis, and IBS calculations with accompanying population structure information supports potentially two mutations, one common to Senepol and Romosinuano and another in Carora, effecting genes contained within our refined location for the *SLICK* locus.

## Introduction

**Figure 1:**
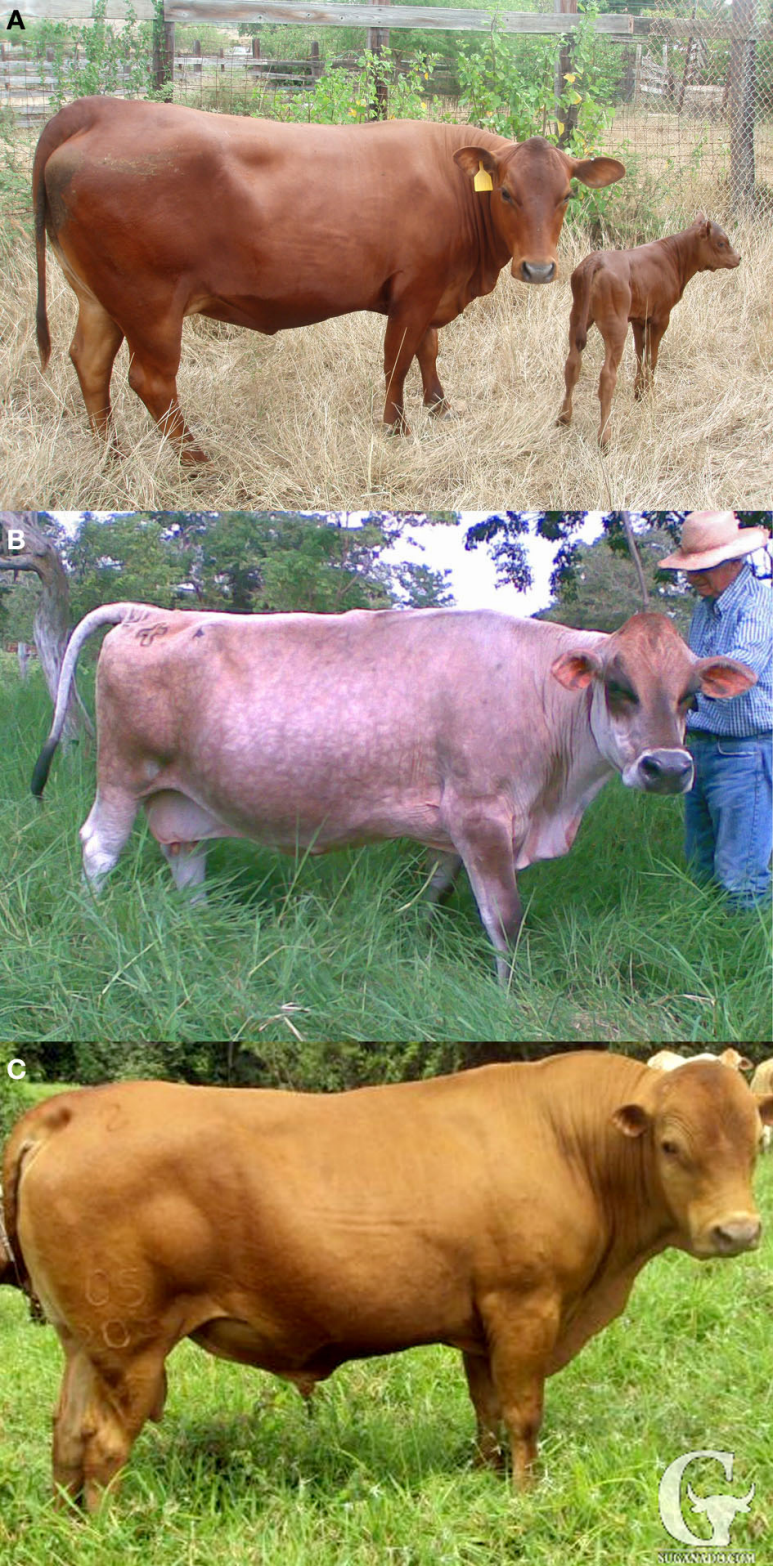
**Tropical slick-haired cattle breeds**. Three Criollo composite cattle breeds **(A)** Senepol, **(B)** Carora, and **(C)** Romosinuano, exhibit the slick hair coat which has been associated with thermo-tolerance in tropical and sub-tropical climates. The slick phenotype is characterized as a fine, sleek hair coat with fewer hair follicles, shorter hair length, and larger sweat glands.

Tropically adapted cattle are known for their ability to tolerate heat stress while maintaining standards of milk yield, reproduction, and disease resistance. The slick hair coat (SLICK) has been attributed as playing an important role in the thermo-tolerance for some adapted breeds being a product of natural selection as well as a modern selection criteria for many breeders in tropical and sub-tropical climates (Olson et al., 2003; Mariasegaram et al., 2007; Landaeta-Hernandez et al., 2011). The SLICK is described as a very short, sleek hair coat mostly observed in tropical *Bos taurus* cattle of Criollo descent in Central and South America as compared to the commonly observed longer hair coat of *Bos taurus* from other climatic zones (Figure 1).

New World breeds characterized as possessing the SLICK include the Senepol, Carora, Criollo Limonero, and Romosinuano (Hammond et al., 1996; Olson et al., 2003; Brenneman et al., 2007; Mariasegaram et al., 2007; Landaeta-Hernandez et al., 2011). Each of these tropically adapted breeds have ancestral roots stemming from Spain during early New World colonization (University, 2008). However, it is not well documented if these Criollo cattle originated from the same or different cattle populations in Iberia (Martinez et al., 2012). Of the slick-haired breeds, Romosinuano were developed in the late 1800's from a line of Costeño con Cuernos Criollo cattle in northern Colombia. The breed possibly has Angus and/or Red Poll ancestry due to their polled nature (University, 2008). Senepol, founded in 1918, are also a composite breed of early Criollo stock from St. Croix crossed to N'Dama and later to Red Poll to obtain an early maturing, docile, polled, beef animal (Hammond et al., 1996, 1998; Olson et al., 2003; Brenneman et al., 2007; Mariasegaram et al., 2007). The Carora originated slightly later in the 1930's by crossing Brown Swiss with north-western Venezuelan Criollo dairy cattle and became a recognized Venezuelan breed in 1985 (Rizzi et al., 2002). In contrast, Criollo Limonero have undergone 500 years of natural selection in northwest Venezuela and have remained in genetic isolation for the past 30 years for breed preservation (Ovillasmil-Ontiveros et al., 2008; Landaeta-Hernandez et al., 2011). The Criollo ancestry provides a common, if tenuous, link among these tropical Central and South American cattle demonstrating the SLICK hair coat.

The importance of thermo-tolerance prompted genetic investigations of the SLICK, which have identified a dominant mode of inheritance and diagnostic markers for the short, sparse hair phenotype (Olson et al., 2003; Mariasegaram et al., 2007). *SLICK* was mapped to a 4.4 Mb region on bovine BTA20 using linkage analysis of cross-bred lineages between slick-haired dams and non-slick sires (Mariasegaram et al., 2007). Two microsatellite markers, DIK2416 and NRDIKM023, were established as diagnostic tools for selective breeding. The *prolactin receptor gene (PRLR)* was suggested as a candidate gene for the slick hair phenotype. More recently, a study of signatures of selection in Senepol using 47 K single-nucleotide polymorphisms (SNP) from Illumina's BovineSNP50 proposed a new candidate gene, *Retinoic Acid induced 14 gene (RAI14)*, for the slick hair phenotype (Flori et al., 2012). While the general region of interest on BTA20 remains the same, the target region continues to shift and the causative mutation remains elusive.

The central aim of this study was to identify new and improved diagnostic markers for the SLICK hair coat and propose a causative mutation. Due to the composite nature of many of the SLICK breeds, we also focused on ancestry analysis for proper use of the dataset and ancestral origin of the trait. To this end, unsupervised cluster analysis and principal component analysis (PCA) were performed to better understand the background of the slick hair phenotype and potential ancestral influence. We utilized a genome-wide association study (GWAS) to narrow the *SLICK* locus by employing a unique balance of slick-haired breeds as cases in comparison to non-slick-haired ancestral breeds as controls. To this end, 195 cattle were genotyped on the Illumina Bovine HD Beadchip generating over 639 K informative SNP markers spanning the bovine genome (Illumina, 2012). This included cattle from slick-haired Senepol, Carora, and Romosinuano, three additional tropical cross-bred lineages, and six ancestral non-slick-haired breeds. We also utilized a novel systematic approach of admixture analysis in an effort to identify potential roles of ancestry influence on the slick phenotype. The results identified 35 associated SNPs and 24 haplotypes, of which three are never present in non-slick individuals and therefore potential diagnostic tools.

## Materials and methods

### Breeds, pedigrees, and phenotype

The slick hair phenotype is a very short, sleek hair coat as compared to the commonly observed longer hair coat of typical *Bos Taurus* (Figure 1). Our study used individuals from three tropical breeds, Senepol (SE), Carora (CR), and Romosinuano (RS), and three tropical cross-bred lines consisting of Senepol × Angus (SNG, SNGSE), Senepol × Holstein (SHO), and Romosinuano × Angus (RAN, ANRAN) which demonstrated the slick phenotype (Table 1). The SHO animals were back-crossed to pure Holsteins over four generations or line-bred between crosses and selected for the SLICK. Overall they ranged from 40 to 97% HO according to pedigree analysis. The SNGSE and ANRAN were also the progeny of back-crossing between SNG and pure Senepol and RAN crossed back to pure Angus. Additionally, individuals of the breeds Angus (AN), Red Poll (RP), N'Dama (ND), Holstein (HO), Brown Swiss (BS), and East African Zebu (ZB), all having non-SLICKs and an ancestral relationship to the slick breeds, were used for comparison in various analyses described throughout the Materials and Methods section. All animals were characterized as having a slick or wild-type hair coat based on visual observations of hair length. The SHO were additionally validated as SLICK using the weight of hair clipped. Senepol, Red Poll, and all three cross-bred lines were validated using SLICK microsatellite markers (Mariasegaram et al., 2007). Biological and phenotypic sample collection followed standard Animal Care and Use protocols established by USDA.

**Table 1:** **One hundred and ninety-five cattle were utilized in the genetic analyses of the slick hair phenotype**.

**Breed code**	**Breed**	**Registered in breed^a^**	**SLICK haplotype^b^**	**Number of individuals^c^**	**Phenotype^d^**	**Notes**
AN	Angus	Yes	No	10	Non-slick	
BS	Brown Swiss	Yes	No	10	Non-slick	
CR	Carora	?	No	10	Slick	
ZB	East African Zebu	No	No	10	Non-slick	
HO	Holstein	Yes	No	7	Non-slick	
ND	N'Dama	Yes	No	10	Non-slick	
ANRAN	RAN × Angus bull	No	Yes	1	Slick	
RP	Red Poll	Yes	Yes	10	Non-slick	
RAN	Romosinuano × Angus	No	Yes	11	Slick	
SE	Senepol	Yes	Yes	69	Slick	2 Individuals non-slick
SNG	Senepol × Angus	No	Yes	5	Slick	1 Individual non-slick
SHO	Senepol × Holstein	No	Yes	38	Slick/non-slick	40–97% HO
SNGSE	Senepol × SNG	No	Yes	2	Slick/non-slick	1 Individual non-slick
RS	Romosinuano	Yes	No	2	Slick	

*This included three slick-haired Criollo composite breeds of Senepol, Carora, and Romosinuano and three cross-bred lineages utilizing Senepol and Romosinuano stock. Six ancestral breeds were included as non-slick control breeds for comparison and breed influence on the slick phenotype*.

a *Individuals used in the study were registered within their respective breed associations*.

b *Microsatellites DIK2416 (53 cM) and NRDIKM023 (54.4 cM) were genotyped or imputed for individuals*.

c *The total number of individuals available for analysis within a breed or cross-breed*.

d *The slick or non-slick phenotype characterized in individuals of each breed or cross-breed wthin the study*.

### Genotyping

Genomic DNA was isolated using standard proteinase K and phenol extraction methods and the QIAGEN Puregene kit for 195 cattle representing nine breeds and three cross-breeds (Table 1). Thirty-seven Senepol were genotyped for SLICK using a commercial microsatellite marker test provided by Neogen (Lincoln, NE). The genotype scores for these two markers, DIK2416 (BTA 20: 37775688–37775824) and NRDIKM023 (BTA 20: 38313942–38314144), were manually reviewed for homozygote or heterozygote inheritance and allowed us to validate the visual observation of the slick phenotype derived from slick haired animals with haired progeny (Mariasegaram et al., 2007). Methods developed by McClure et al. were used to assign microsatellite haplotypes correspondimg to the haplotypes derived from high-density SNP genotypes (Mcclure et al., 2012). These individuals were used as a training set to impute SLICK inheritance from the Illumina BovineHD Beadchip genotypes (Illumina, 2012). All 195 cattle were genotyped on the Illumina BovineHD Beadchip for an in-depth genome-wide investigation of the slick phenotype. Genome Studio software was used to call and cluster over 777 thousand SNP marker genotypes from BovineHD (Illumina, 2012). Quality control measures using PLINK software retained over 639 thousand SNPs having greater than 90% genotyping call rate and greater than 5% minor allele frequency. All individuals having over 95% genotype call rate were retained (Purcell et al., 2007). The UMD 3.1 bovine genome assembly was used for establishing coordinates (Kent et al., 2002; Karolchik et al., 2014; UCSC, 2013).

### Genome-wide association study

GWAS were run using 639,663 SNPs in a case/control analysis approach on EMMAX software which corrects for population stratification and relatedness (Kang, 2010). Significance levels were generated using basic (adaptive) permutation testing in PLINK (Purcell et al., 2007). The most highly associated SNPs (Supplemental Table 1) had a permutated *p*-value less than 1 × 10^−6^ at 1,000,000 permutations. The least related individuals from each lineage were chosen for the GWAS based on identity-by-descent (IBD) estimations produced by Golden Helix SVS software due to the lack of pedigrees for some individuals and the convoluted line-breeding in the cross-breed pedigrees (Golden_Helix, 2012). Linkage disequilibrium (LD) was calculated using the expected maximization algorithm (EM) to derive *r*^2^ estimates of pairwaise LD using the Golden Helix SVS software. The key GWAS utilized 72 slick cases including 36-SE, 3-SNG, 1-SNGSE, 1-ANRAN, 11-RAN, 8-SHO, 10-CR, and 2-RS. This was balanced with 61 non-slick control animals including 2-SE, 1-SNG, 1-SNGSE, 10-RP, 10-ND, 10-ZB, 10-AN, 7-HO, and 10-BS. Due to the nature of breeds such as the Senepol and Carora being predominantly slick coated, ancestral breeds such as the Red Poll, N'Dama, and Brown Swiss were used to serve as non-slick control animals. We also included East African Zebu cattle as a control breed based on recent population modeling of Senepol cattle (Flori et al., 2012). Non-slick coated Angus and Holstein were used as controls to counter the slick coated cross-breed lineages. We hoped to minimize population stratification and reduce false SNP association to breed specific alleles by taking this approach. In a second GWAS, we removed the Carora cases and Brown Swiss controls and in a third GWAS, we removed the Romosinuano, RAN, ANRAN, and Angus. The systematic removal of slick breeds and their ancestral groups was to identify any shifting of the GWAS peak location and the subsequent effect on the *p*-value of associated SNPs.

Haploview software was utilized to identify significantly associated haplotype blocks with respective allele identification (Barrett et al., 2005). Significance levels were generated using permutation testing within Haploview software using 100,000 permutations and a permutated *p*-value threshold of less than 0.001. Block designation and numeric ID were generated in Haploview with specific blocks referenced by their numeric ID hence forth. A Case/Control association test was used ignoring pairwise comparisons of markers when greater than 1000 kb apart.

### Population structure

We ran PCA and unsupervised clustering algorithms to better understand the background of the slick phenotype in regards to breed and ancestral influence. PCA was performed using EIGENSTRAT methodology embedded in the Golden Helix SVS software (Patterson et al., 2006; Price et al., 2006; Golden_Helix, 2012). Model-based unsupervised clustering based on maximum likelihood population estimations was performed using the software ADMIXTURE (Alexander et al., 2009; Alexander and Lange, 2011). The optimal K-value within ADMIXTURE was identified with the inferred number of populations producing the lowest cross-validation error which is estimated during the clustering analysis. Both PCA and ADMIXTURE analyses were examined using the 133 individuals included in the GWAS panel. ADMIXTURE analysis was broken down in a step-wise fashion first using the 639,663 SNPs dispersed genome-wide. Subsequent ADMIXTURE analyses examined the same GWAS individuals on BTA20 by utilizing 19,475 SNPs spanning the entire chromosome and then narrowing our region of interest further to an 18 Mb (29544031–47548079 bp) region, ~30% of chromosome 20, within and surrounding our target area and utilizing 4351 SNPs. The step-wise reduction in genomic regions for population clustering identified variation in admixture fractions within slick individuals and the major breed signatures found in SLICK cattle. Comparable PCAs were run on the same 4351 SNPs, comprising 30% of BTA20, all of BTA20, and genome-wide.

We also conducted a more refined examination of population structure within the Senepol breed. ADMIXTURE analyses were run genome-wide, on BTA20, and on 30% of BTA20 utilizing 38 Senepol and 10 of each referenced Senepol ancestral breed including N'Dama, Red Poll, and East African Zebu. Additionally, we included Romosinuano cattle due to their potentially similar Criollo and Red Poll background which was supported by preliminary cluster analysis observations. A comparable ancestral analysis was not run on the Carora cattle due to lack of more than one ancestral reference breed being available for genotyping.

### Runs of homozygosity

We examined runs of homozygosity (ROH) in 130 slick-haired individuals on BTA20 representing the effect of recent autozygosity in a genomic region (Mcquillan et al., 2008). Custom Perl scripts were written for this analysis with PLINK software confirming the results. Considering unknown relatedness between individuals, ROH was defined by various thresholds with 100, 200, 300, and 500 consecutive homozygous SNPs, which is approximately corresponding to regions of 0.3, 0.6, 1.0, and 1.6 Mb, respectively. The sum of ROH state (1 or 0) of each SNP was divided by the total number of slick-haired animals to calculate the frequency of ROH for each locus. These results summarized local autozygosity produced by the recent mating of related animals.

### Signatures of selection

To assess the selection of the slick phenotype in the resource population, haplotypes were inferred with fastPHASE (Scheet and Stephens, 2006) for each breed, and evidence for positive selection was detected by calculating the value of the standardized integrated extended haplotype homozygosity (|iHS|) for each SNP across BTA20 as described by Voight et al. (2006). Due to high consanguinity among slick-haired animals, we scanned extended haplotypes using 22,000 loci with a boundary up to 5 Mb from the core position. The average |iHS| score for each locus was used to reduce background signals unrelated to recent selection in regard to the slick hair phenotype. Alleles having a higher frequency in slick animals were considered an ancestral type allele. The standardized |iHS| value on BTA20 was obtained using rehh package in R (Voight et al., 2006; Gautier and Vitalis, 2012).

### Identity by state of haplotype

For the purpose of narrowing the map position of the *SLICK* locus, we examined consensus haplotypes bound to the slick phenotype in each cattle breed. We then compared slick haplotypes between breeds using 5 SE, 2 SHO, and 5 CA, all of which were homozygous individuals across the *SLICK* region. In this analysis, the haplotype on chromosome 20 was partitioned with a 1–50 SNP window frame to search for identical haplotypes within slick-haired Senepol, Carora, and the SHO crossbred group. When considering the ratio of slick-haired animals, frequency of a SLICK identity by state (IBS) haplotype is anticipated to be 0.4 or higher within a breed due to the dominant heritability. Slick-haired animals were expected to show a similar haplotype based on a common origin from only a few related shared ancestors.

## Results

### Genome-wide association study and linkage disequilibrium

**Figure 2:**
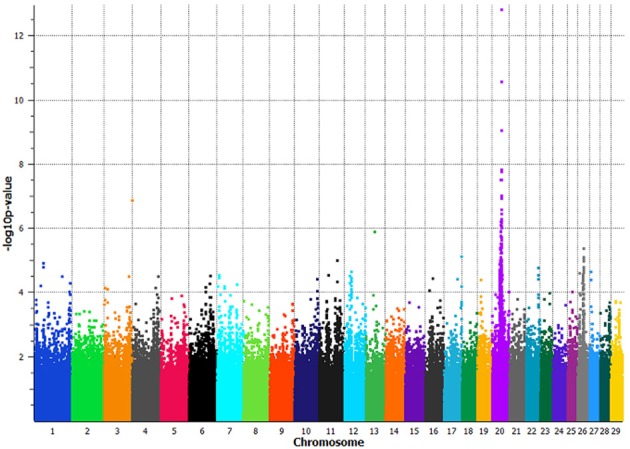
**Manhattan plot of a genome-wide association study comparing slick-haired tropical breeds and cross-bred lineages to non-slick-haired ancestral breeds**. Seventy-two slick-haired animals from the tropical breeds of Senepol, Carora, Romosinuano and cross-bred lineages were compoared to 61 non-slick animals from Red Poll, Brown Swiss, Holstein, Angus, N'Dama, and East African Zebu ancestral breeds. Non-slick individuals from the cross-bred lineages were included with the ancestral control breeds. Significant association of the slick phenotype was found on BTA20 at 38.5 Mb with the peak SNP having a *p*-value of 1.54 × 10^−13^ using a panel of 639,663 SNPs spanning all bovine autosomes. The x-axis denotes chromosome 1 through 29 with SNP positions plotted in increasing genomic order. The y-axis plots the −log^10^ *p*-value as determined in an association analysis using the program EMMAX.

The GWAS utilizing the full dataset of 133 least related individuals, had a maximum −log^10^ *p*-value of 12.81 with a corresponding *p*-value of 1.54 × 10^−13^ on BTA20 at 38,544,165 bp, *BovineHD2000010982* (Figure 2). In total, 35 SNPs spanning 4.4 Mb on BTA20 (34,928,482–39,345,284 bp) had *p*-values less than 9 × 10^−7^ (permutated *p*-values < 1 × 10^−6^ at 1,000,000 iterations) (Supplemental Table 1). Linkage disequilibrium (*LD* = *r*^2^) increased 7-fold, 10-fold, and over 17,000 fold in a pair-wise comparison of the three most significantly associated SNPs between non-slick versus slick-coated individuals (Supplemental Table 2). The associated region was decreased to 2 Mb (37,179,958–39,345,284 bp) with the removal of a single SNP at 34,928,482 bp which was in moderate LD with the principle associated SNP.

The removal of the Carora and Brown Swiss breeds decreased the association significance to a *p*-value of 1.53 × 10^−10^ and moved the peak SNP 500,000 base pairs upstream to a location of 38,031,125 bp, *BovineHD2000010877*. This SNP was not significantly associated in the full GWAS animal panel.

The removal of the pure Angus and Romosinuano cattle and the RAN lineage decreased the association to a *p*-value of 7.48 × 10^−12^ and shifted the peak associated SNP upstream to a location of 38,112,689 bp, *BovineHD2000010897*. This SNP was the second most significantly associated marker in the full GWAS animal panel (*p*-value 4.37 × 10^−11^).

Twenty-four allelic patterns encompassing 22 haplotype blocks on BTA20 were significantly associated (permutated *p*-values <0.001; 100,000 permutations) (Table 2). These haplotype blocks started at 35,029,690 bp and ended at 40,588,890 bp. A total of 21 allelic patterns were associated with slick coated cattle and three associated to non-slick coated individuals. Blocks 94 and 137 had two different allelic patterns in association, one pattern associated with slick coated and the other with non-slick coated animals. Block 53 was the shortest block consisting of 2 consecutive SNPs, 1.9 kb apart. Block 138 was the longest block at 55.9 kb in length and containing 19 SNPs. Blocks 104, 112, and 143 were haplotypes only found in slick-haired individuals. Block 94 (GGG) had the largest increase in frequency at 50% from non-slick (0.314) to slick (0.833) individuals.

**Table 2:** **The 24 most highly associated haplotype patterns on BTA20 for the slick/non-slick phenotype were identified and given a Block ID**.

**Block ID**	**Start bp**	**End bp**	**Block distance (bp)**	**Haplotype**	**Total frequency**	**Frequency SLICK**	**Frequency non-SLICK**	***P*-value**
2	35029690	35073431	43,741	AGACGACG	0.126	0.292	0.025	8.39E-08
52	36293763	36306435	12,672	AAGAAAG	0.111	0.264	0.017	1.39E-07
53	36311419	36313371	1952	CC	0.191	0.389	0.071	6.15E-08
58	36396764	36441238	44,474	GCAAAGGGAGGGGGGGGCGGGGCAA	0.105	0.264	0.008	2.62E-08
87	37534901	37572302	37,401	GCAAGGAGCAAAG	0.131	0.29	0.034	3.66E-07
94	37718791	37721846	3055	GGG	0.511	0.833	0.314	3.58E-12
94	37718791	37721846	3055	AAA	0.453	0.153	0.636	8.80E-11
100	37834790	37847125	12,335	GGGCGA	0.184	0.389	0.059	1.31E-08
102	37885136	37935057	49,921	GCAGAGGCGGAGAAGG	0.116	0.292	0.008	3.25E-09
104	37940179	37957238	17,059	GGGGA	0.111	0.292	0	4.96E-10
110	38182103	38220127	38,024	GCAAGG	0.119	0.289	0.015	1.59E-08
112	38224054	38281493	57,439	GGGGAGG	0.105	0.278	0	1.44E-09
117	38384676	38412185	27,509	AAGAGGAGA	0.226	0.444	0.093	1.99E-08
118	38418823	38440030	21,207	GGGGG	0.658	0.903	0.508	2.73E-08
119	38446577	38523760	77,183	GAGAAAAGAGAG	0.138	0.306	0.035	1.58E-07
120	38539025	38550947	11,922	CAG	0.474	0.167	0.661	3.58E-11
125	38878307	38896758	18,451	AAGGAAGAG	0.742	0.958	0.61	1.03E-07
131	38975737	39004699	28,962	AAG	0.531	0.763	0.39	5.45E-07
137	39262981	39267121	4140	AAAG	0.5	0.764	0.339	1.33E-08
137	39262981	39267121	4140	GGGA	0.489	0.236	0.644	4.84E-08
138	39270125	39326031	55,906	AGAAAGAACGAGAGGAACA	0.474	0.75	0.305	2.55E-09
139	39337264	39367991	30,727	CCGGGAAAAGCGA	0.121	0.278	0.025	2.30E-07
143	39469953	39508807	38,854	GGGAGGGCAGCGGGAGGAGA	0.1	0.264	0	4.06E-09
179	40558060	40588890	30,830	AGAAG	0.405	0.681	0.237	1.56E-09

*Specific location, length, and haplotype with allele designation are identified for each block with frequency calculations of said haplotype within slick and non-slick individuals. Blocks 104, 112, and 143 were found only in slick individuals and block 94 (GGG) had the greatest increase in frequency from non-slick to slick individuals therefore marking these haplotype patterns as the best candidates for diagnostic markers*.

### Population structure

**Figure 3:**
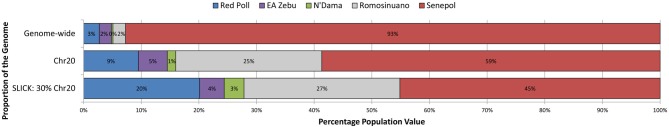
**The variation in average genetic breed composition of Senepol cattle using targeted unsupervised clustering analysis**. Variation in genetic breed composition was investigated in 38 Senepol cattle to ascertain the possible role of ancestry influence on the slick hair phenotype within this breed. In a step-wise fashion, breed composition was compared on a genome-wide level (top), using BTA20 (middle), and targeting the *SLICK* locus (bottom). The percentage of each genetic breed signature is denoted by a different color. The Senepol generated a unique signature denoted in red, with blue representing Red Poll, purple being East African Zebu, green being N'Dama, and gray being Romosinuano.

Unsupervised clustering analysis and PCA showed population structure across our study animals and insight of potential ancestral lineages contributing to the slick phenotype. Genetic patterns identified in ADMIXTURE analysis aligned with breed definition of individual animals, therefore ADMIXTURE populations are referred to by the dominating representative breed. The optimal *K*-value in the analysis of Senepol ancestry was *K* = 5 where Senepol and all four other breeds utilized formed distinct breed clusters based on genetic signatures of allele frequency. Genomic verification of Senepol ancestry utilizing ADMIXTURE confirmed genomic components of Red Poll, N'Dama, and East African Zebu (Supplemental Figure 1A,B). Senepol also shared a genetic component with Romosinuano cattle. An average breed composition of Senepol cattle consisted of 93% unique Senepol genetic signature, 3% Red Poll, 2% East African Zebu, 2% Romosinuano, and less than 1% N'Dama breed signature using genome-wide SNP data (Supplemental Figure 1A). We note the potentially inflated Senepol breed signature due to this breed having nearly 4-fold more individuals representing the breed as compared to each ancestral breed. Variation in Senepol breed composition was identified in a comparison of genome-wide, BTA20, and 30% of chromosome 20 admixture analyses (Figure 3). The genetic signature distinct to Senepol decreased from 93% on the genome-wide analysis to 45% when the analysis targeted the *SLICK* locus. The larger percentage of Senepol signature in the genome-wide analysis is potentially a by-product of their unique composite breed pattern being identified across the entire genome as compared to only a fraction of this 93% can be identified in the much smaller *SLICK* region.

**Figure 4:**
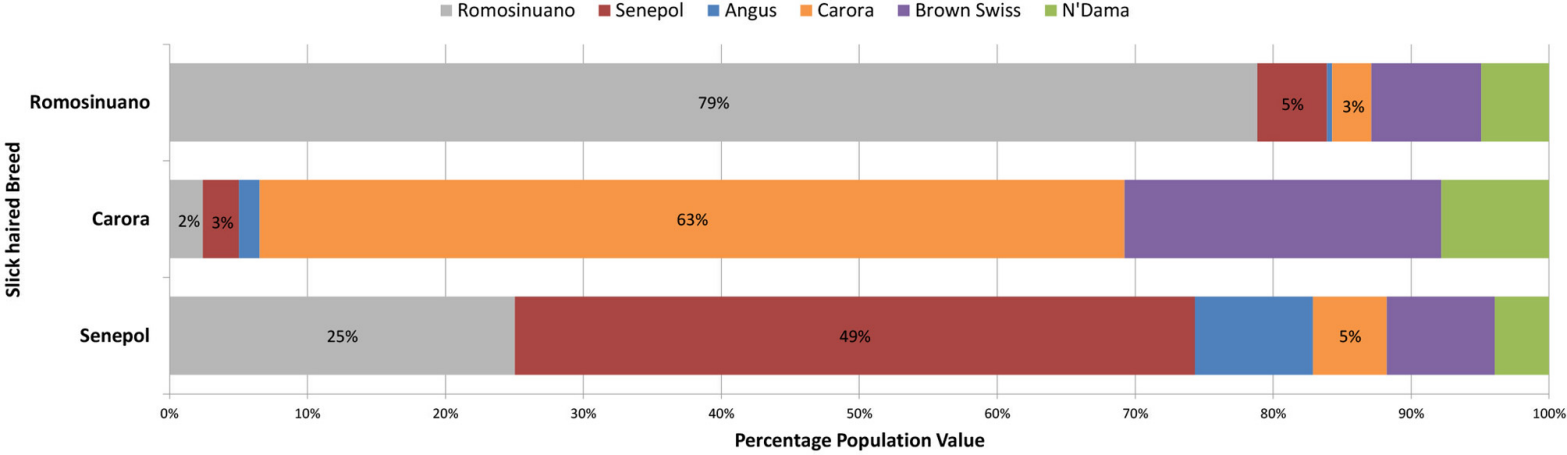
**The average genetic breed composition of Romosinuano, Carora, and Senepol when targeting *SLICK***. The three tropical slick-haired breeds were compared for their average breed composition using the ancestral breeds of Angus, Brown Swiss, Red Poll, N'Dama, and East Afrian Zebu as well as the three tropical breeds themselves to identify variation and relationship between the breeds at *SLICK*. At the optimal population differentiation value of *K* = 6, the Red Poll and East Afrian Zebu signatures were not identified. The percentage of each remaining genetic breed signature is denoted by a different color. The Romosinuano are denoted in gray, Senepol in red, Angus is blue, Carora in orange, Brown Swiss in purple, and N'Dama in green. The Senepol demonstrate a stronger genetic similarity to Romosinuano than Carora but all three share some degree of similarity to one another and ancestral breeds.

A traditional whole-genome analysis of admixture within and among the breeds utilized for the GWAS including the 6 lineages represented in Table 1, provided a foundation for breed relationship and insight into ancestry of the composite breeds. ADMIXTURE results utilizing 639,663 SNPs across the entire genome showed clear population structure correlating to continental origin and breed relationship. The first separation of a population was comprised of Senepol cattle. However, we note the formation of this population might be biased due to this breed having nearly 4-fold more individuals than the other breeds. We chose to allow this difference in order to investigate the individual breed composition of the multiple slick coated Senepol in our analyses. At *K* = 3, or 3 populations, the African breeds N'Dama and East African Zebu clustered together. The Carora and their ancestor breed, Brown Swiss, clustered together at *K* = 4. East African Zebu and N'Dama separated into distinct populations at *K* = 5 which was the optimal *K*-value based on ADMIXTURE's cross-validation error rates. At 6 populations, both the purebred Holsteins and the cross-bred SHO form a cluster. Red Poll cattle became a distinct cluster at *K* = 7, and Romosinuano followed at *K* = 8. A comparison of breed composition between the Senepol, Carora, and Romosinuano at the optimal *K* = 6, shows varying measures of genetic similarity based on each breed's genetic signature (Figure 4). In total, each of the slick-haired breeds has a genetic component specific to the other two slick-haired breeds when targeting the *SLICK* locus.

ADMIXTURE analysis of these same individuals using 19,475 SNPs spanning chromosome 20 provided a comparison of breed relationship and ancestry when targeting a specific area of the genome. This analysis showed an overall clustering pattern where populations were less defined, with individuals having lower clustering scores within populations and higher levels of admixture. The same order of breed and continental population structure followed for the full SNP dataset analysis pattern was followed for ADMIXTURE analysis using 4351 SNPs spanning 18 Mb, ~30% of the chromosomal region, surrounding our target *SLICK* locus on BTA20. The Senepol breed signature in the SHO and RAN crosses linked the majority of the slick phenotyped individuals as the analysis targeted the *SLICK* locus. Carora, one of the slick-haired breeds, showed little common population structure with any of the other slick-haired breeds. In contrast to Senepol where their genetic breed signature decreased within the breed as analysis was narrowed to the *SLICK* region, the Carora breed signature increased. The average percentage of Brown Swiss genomic composition within Carora declined from 84 to 71 to 56% respectively in genome-wide, chromosome 20, and 30% of BTA20 analyses.

Principal component analyses corroborated the basic findings of ADMIXTURE population structure previously outlined (Supplemental Figure 2). Senepol and the African N'Dama and East African Zebu formed the most distinct breed clusters in the genome-wide analysis (Supplemental Figure 2A). Chromosome 20 ADMIXTURE analysis most clearly separates the slick coated lineages, excluding Carora, from the non-slick breeds (Supplemental Figure 2B). The breed clusters become less defined with ADMIXTURE analysis of the *SLICK* locus but retain a basic separation of the slick from the non-slick cattle (Supplemental Figure 2C).

### Runs of homozygosity and signatures of selection

**Figure 5:**
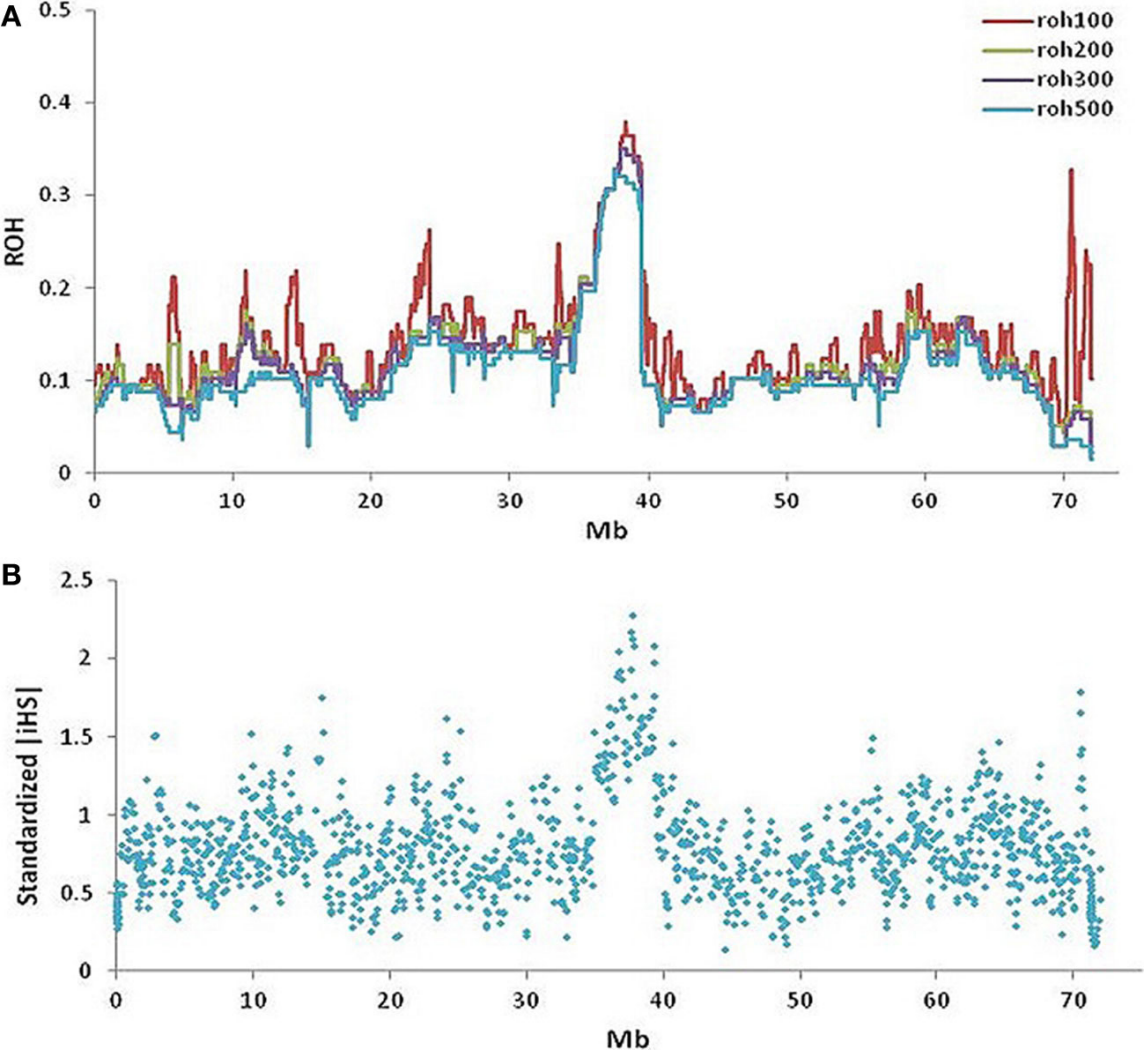
**Runs of homozygosity and signatures of selection on BTA20 within slick-haired animals. (A)** Runs of homozygosity using windows of 100-red, 200-green, 300-purple, and 500-blue SNPs (~0.3, 0.6, 1, 1.6 Mb respectively) showed a peak value of 0.4 between 37.5 and 39.5 Mb on BTA20 within slick-haired animals. **(B)** Standardized |iHS| was used to determine signatures of selection in a comparison of slick to non-slick individuals using a 30 SNP window frame across BTA20. The peak |iHS| value was at 37.7 Mb on BTA20.

The maximum autozygosity among 130 slick-haired individuals was 0.4 in the region between 37.5 and 39.5 Mb (Figure 5A) on BTA20. Heterozygosity was determined as ~0.7 in Senepol and 0.6 in Carora at the *SLICK* locus, consistent with an expected frequency of *SLICK* alleles based on pedigree analysis. The most common haplotype had a relatively high frequency of 0.6 presumably due to selection and introgression. A comparison of ROH between Senepol and Carora, which are not directly related breeds, allowed us to define the probable interval spanning the *SLICK* locus.

Standardized |iHS| revealed 1211 loci having an |iHS| score greater than 2, implying these regions were subject to recent selection (Figure 5B). The highest average |iHS| value was at 37.7 Mb on BTA20. This region overlaps the region identified in the GWAS.

### Identity by state of haplotype

**Figure 6:**
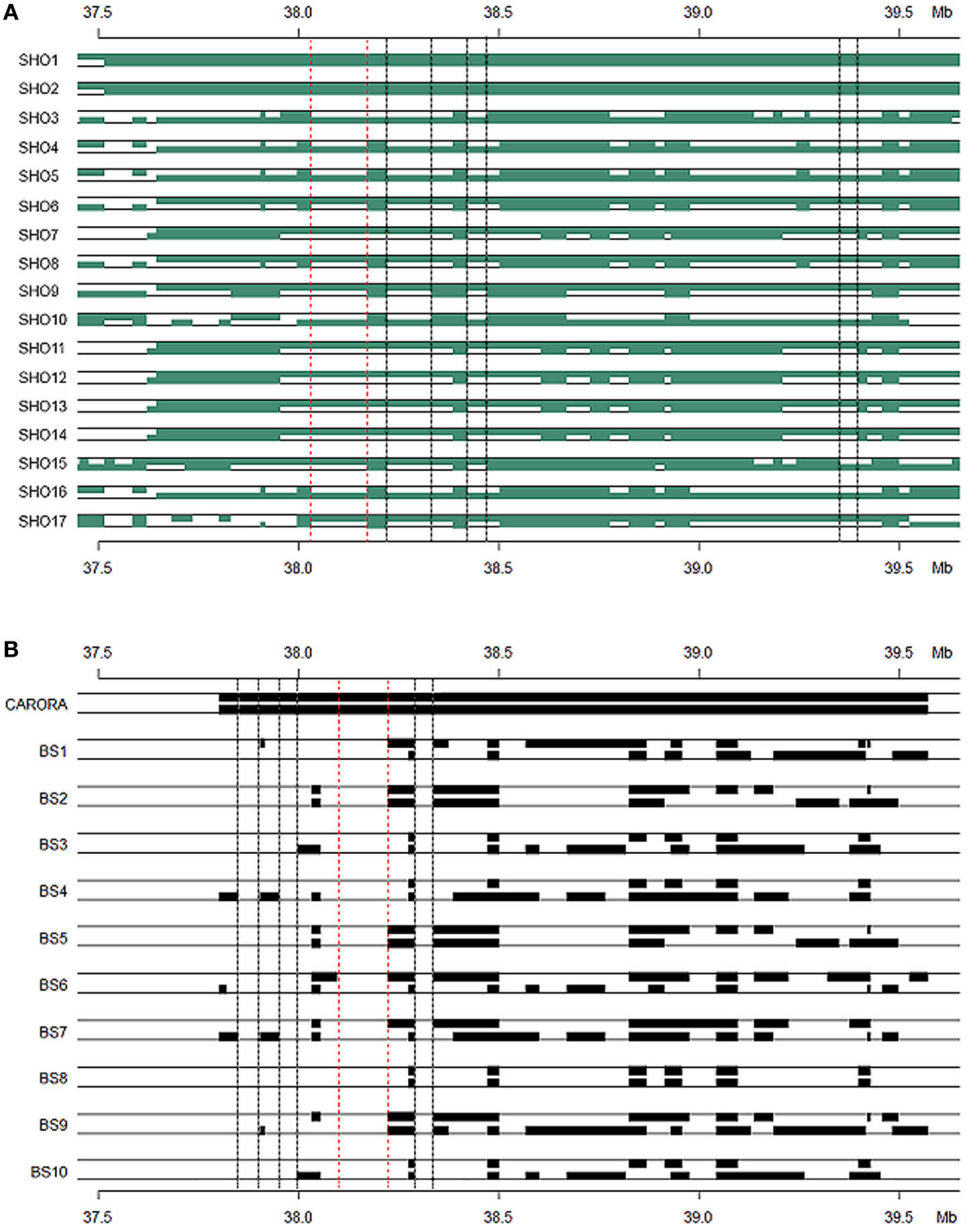
**Haplotype blocks of Senepol-Holstein cross-bred and Caroro/Brown Swiss cattle based on identity by state analysis**. Animals expected to be heterozygous at *SLICK* are shown with each block representing a common haplotype greater than ten SNPs. The dotted lines indicate consensus regions among the slick-haired animals potentially containing the *SLICK* mutation. The region between the red dotted lines are the most likely candidate regions with those between black dotted lines being less likely according to phenotype. The y-axis denotes genetic position on BTA20 in Mb. **(A)** Senepol-Holstein individuals are denoted on the x-axis with the first two animals being homozygous across SLICK. The most probable candidate region lies between 38.0 and 38.2 Mb. **(B)** A comparison between the homozygous region in Carora as shown by the first individual and opposing non-slick Brown Swiss as the remaining individuals. The most probable candidate region lies between 38.1 and 38.2 Mb.

The Senepol × Holstein (SHO) crossbred line was our initial target group to identify regions of common IBS haplotype of the slick phenotype. The cross breeding of this line between the slick coated Senepol and the non-slick Holstein would have the effect of breaking the ancestral blocks derived from Senepol into smaller regions through recombination. Therefore we expected *SLICK* to be in a smaller consensus haplotype block within the cross-bred lineage, hence narrowing and confirming our region of interest. To this end, two SHO individuals, SHO1 and SHO2, shared an identical haplotype, which was validated by ROH, between 37.5 and 39.5 Mb (Figure 6A). This haplotype block (>2 Mb) was found in the homozygous state within five additional Senepol cattle and no Red Poll, therefore confirming haplotype ancestry origin. IBS haplotype was investigated in the remaining SHO individuals with consensus lengths of region (100–500 kb) being observed in the 17 slick coated SHO individuals. The heterozygous nature of the SHO reduced the candidate region from 1.5 Mb to potential regions of a couple hundred kilobases. Figure 6A displays the consensus IBS haplotype regions likely to possess the *SLICK* locus between 38.0–38.3 Mb (red dotted line: most likely), 38.4–38.5 Mb, and 39.3–39.4 Mb (pink dotted lines: less likely).

Using the same method described above, we found an IBS haplotype corresponding to the slick phenotype in Carora, which extended from 37.3 to 39.5 Mb (Figure 6B). We used the defined region in Senepol to set boundries and then scanned IBS haplotype to find regions of differentiation between Carora and their non-slick Brown Swiss ancestral lineage. The largest common fragment between Carora and Brown Swiss barely exceeded 200 kb presumably as a consequence of the differentiation of two breeds initiated several decades ago. Although short intact haplotypes (50 kb~200 kb) have been conserved, Carora animals have exclusive haplotypes in 4 regions (>20 kb), in particular 120 kb fragment between 38.09 and 38.22 Mb (Figure 4B). The general IBS candidate region in Carora extended from 37.8 and 38.3 Mb.

Lastly, consensus IBS haplotype blocks were identified between slick-haired Senepol, Carora, and the SHO lineage (Supplemental Figure 3). Only very small haplotype fragments of 20 consecutive SNPs or less were conserved between the Senepol and Carora. The two overlapping haplotype blocks using 20 SNP windows fell between 38.8–39 and 39.4–39.5 Mb with multiple smaller (5,10, 15 SNP) haplotype blocks identified within the target region. The two 20 SNP blocks did not coincide with the suggested *SLICK* boundries of 37.8–38.3 Mb identified through within breed IBS analysis (Figure 6) as expected.

## Discussion

Breeds of tropical cattle such as the Senepol, Carora, and Romosinuano have adapted to warmer climates through natural selection toward a short, fine, sleek hair coat, referred to as the slick hair phenotype, that allows them to maintain lower body temperatures under heat stress (Olson et al., 2003). Specifically, slick-haired Criollo Limonero exhibit fewer hair follicles and shorter hair than *Bos indicus* as well as increased sweat gland size and high blood flow irrigating the skin (Landaeta-Hernandez et al., 2011). Modern industry seeks to monopolize on this thermo-tolerance by breeding slick-haired cattle, specifically Senepol, into Holstein bloodlines for the purpose of generating high producing Holstein × Senepol hybrids with greater thermo-tolerance. Here, modern genomic tools are utilized to perform a multitude of analyses narrowing and validating the locus controlling the slick hair phenotype. We identify variation of ancestral lineages within slick-haired breeds such as the Senepol, Carora, and Romosinuano and establish a consensus locus of 0.8 Mb in length (37.7–38.5 Mb) on BTA20 through genome-wide association (GWA), haplotype analysis, signatures of selection, ROH, and IBS calculations.

Analysis of population structure utilizing unsupervised clustering in ADMIXTURE and PCA confirmed Senepol ancestral relationships to Red Poll, East African Zebu, and N'Dama. The Senepol had a greater percentage of genetic similarity contributed to Red Poll with successively less similarity attributed to the East African Zebu and N'Dama respectively. This aligned with (Flori et al., 2012) analysis of Senepol showing predominantly European Taurine origins (89%), with Zebu (10.4%), and African Taurine (0.6%) comprising lesser ancestral contribution respectively (Flori et al., 2012). Using the same clustering value of *K* = 3 as in the Flori study, Senepol averaged 94.7% Red Poll, 4.4% East African Zebu, and 0.8% N'Dama ancestry for our study.

Shared genetic patterns were evident between each of the three slick-haired breeds with greater similarity between Romosinuano and Senepol (Figure 4 and Supplemental Figure 1). On average, Senepol had the highest similarity, 25%, to Romosinuano. Carora had the lowest similarity to either breed at 2% Romosinuano and 3% Senepol. Carora segregated separately from any of the slick-haired individuals on PCA analysis (Supplemental Figure 1) whether comparing at a genome-wide level or at *SLICK*. Carora consistently clustered with Brown Swiss demonstrating a strong genetic similarity to their ancestral breed. Regardless of the degree of genetic similarity shared between each of the slick-haired breeds, it is unknown as to which genetic breed signature is representative of the slick phenotype itself. A key missing element to ancestry analysis of the slick phenotype origin is that of the Criollo breeds. Criollo ancestry is the common link between origination of each of these composite tropical breeds. The Criollo Limonero, as an isolated slick-haired Criollo breed with no cross-mating, may give future insight as to the ancestral origin of the slick hair phenotype.

**Figure 7:**
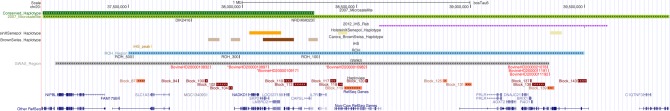
**Comparison of BTA20 regions associated with the SLICK hair phenotype derived from different genetic analyses**. Regions of association are highlighted from prior research of the SLICK hair phenotype and compared to the results for this study within an annotated UCSC Genome Browser window. The browser window encompasses genome coordinates from UMD 3.1 on BTA20:37–40 Mb. The top two green lines depict the associated region (light green) and conserved haplotype (dark green) from 2007 (Mariasegaram et al., 2007). The microsatellites, DIK2416 and NRDIKM023 are identified just below as black lines and labeled. The signature of selection identified by (Flori et al., 2012) is shown in purple. The conserved haplotype regions within the SLICK Holsteins with Senepol admixture and Carora (Brown Swiss × Criollo) are shown in orange and brown respectively with the most significant of these regions in the darker shaded colors. Run of homozygosity are depicted in blue with peak regions for the 500, 300, and 100 SNP windows labeled just below. Genome-wide association is depicted in gray with the most significantly associated SNPs labeled below in red. SNPs BovineHD200010897, BovineHD2000010917, and BovineHD2000010982 showed the highest association and defined the 0.5 Mb region of interest. Lastly, haplotype blocks in association with the SLICK phenotype are identified in brick red under the GWAS SNPs, and those blocks with the most significant associations being more darkly shaded. Annoted bovine gene and other refseq genes are shown at the bottom of the figure (dark blue).

While the investigation of population structure showed variations in ancestry and genetic similarity among the slick-haired breeds; GWA, haplotype analysis, signatures of selection and IBS calculations were performed to narrow *SLICK* and produce potential diagnostic markers for the phenotype. In previous studies, the 4-cM interval, equivalent to ~2 Mb region, containing *SLICK* comprises roughly 20 genes (Mariasegaram et al., 2007). Recent signatures of selection identified in Senepol by the Flori group (Flori et al., 2012) demarcate broad regions around *SLICK* due to long extended haplotypes which again includes a number of candidate genes to be examined. Figure 7 overlays Mariasegaram et al.'s original linkage study in 2007, the Flori et al. study from 2012, and each of our analyses onto the UCSC Genome Browser with annotated genes. This highlights the board overlap of regions while showing variation in specific target areas depending upon analysis method used (Mariasegaram et al., 2007; Flori et al., 2012; UCSC, 2013). Our GWAS employed a novel approach of comparing slick-haired “case” animals which were predominantly composite breeds and cross-bred lineages to non-slick-haired “control” animals from ancestral or founding breeds of the respective composites. This allowed for the use of an association study as opposed to linkage analysis by adding non-slick control individuals to the very few non-slick Senepol, Romosinuano, and Carora. The effects of population structure and relatedness were minimized by using individuals from ancestral breeds, identity by descent calculations to remove the most related individuals, and utilizing a kinship matrix in the GWAS. A target region of less than 0.5 Mb (37.7–38.5 Mb) was identified by the three most significantly associated SNPs (*p*-values from 1.54 × 10^−13^ to 8.87 × 10^−10^) (Supplemental Table 1). In addition, three significantly associated haplotype patterns were identified at 0 frequency in non-slick individuals. A combination of highly associated SNPs and haplotype patterns could potentially lead to a new diagnostic marker panel for the slick phenotype.

As an alternative to using a GWA approach, IBS haplotypes were analyzed on BTA20 in the Senepol and cross-bred SHO (Figure 6A). Recombination events occurring within the cross-bred SHO lines broke down the longer haplotype blocks associated with Senepol into smaller blocks potentially containing *SLICK*. In comparison to Carora, a 200 kb region overlapping the Senepol and Carora IBS haplotypes was defined between 38.1–38.2 Mb (Figure 6B). When combining the two breeds for IBS analysis, only small (<20 SNPs) haplotype blocks were identified suggesting the breeds have had no common ancestor for hundreds of generations. In addition, signatures of selection and ROH validated the GWAS and IBS target region with peak associations at 37.7 and 38 Mb respectively (Figure 5). We found the long range haplotype homozygosity (ROH ~0.4) extended over 5 Mb from *SLICK* in slick-haired cattle, which is consistent with the regions bound to recent selection (Flori et al., 2012). The |iHS| analysis, which utilizes decay of haplotype, provided limited mapping resolution due to the same long range haplotype blocks. It is important to note that within this region, two genes, *SPEF2* (38.4 Mb) and *PRLR* (39.0 Mb), corresponding to reproduction and milk production, may have also been targets of natural and artificial selection. Interestingly, no coding region was found between *SPEF2* and *PRLR*, whereas values of |iHS| indicated that the region between *SPEF2* and *PRLR* is substantially subject to recent selection. Six genes are annotated in the bovine genome with an additional four genes annotated in comparative reference genomes within the 0.8 Mb *SLICK* that our study identified. This locus is upstream ~0.5–0.8 Mb from the previously suggested candidate genes of *PRLR* (Mariasegaram et al., 2007) and *RAI14* (Flori et al., 2012). However, a shared IBS 20 SNP haplotype block (Supplemental Figure 3) and three associated allelic patterns (Table 2) cover regions of these genes. Disparities in IBS haplotype blocks where non-slick-haired individuals shared common haplotypes to those carried by the slick individuals made *PRLR* and *RAD1* genes less likely candidate genes for this study. Instead, the *S-phase kinase-associated protein 2*, or *SKP2*, is our primary candidate gene for the slick phenotype based on the consensus region and literature review of the gene. Multiple studies have demonstrated that *SKP2* is a negative regulator of the CDK inhibitor, *p27*^*Kip*1^ (Sistrunk et al., 2011; Kullmann et al., 2013). In particular, the degradation of *p27^Kip1^* by *SKP2* has been documented as a major factor in normal keratinocyte proliferation and skin homeostasis (Sistrunk et al., 2011). Varying levels of *p27^Kip1^*are correlated to hair-fiber thickness and shape in mice suggesting that *SKP2* might be a participant in this network influencing the number and size of keratinocytes which is highly correlated to hair follicle development (Sharov et al., 2006). Both IBS and GWAS evidence supports *SKP2* as a candidate gene for the slick phenotype. IBS identified 10 haplotypes within the *SKP2* gene of Senepol, of which two haplotypes appropriately segregated among the slick-haired heterozygous SHO individuals. Furthermore, one of the three significantly associated GWAS SNP is located in the *SKP2* gene with LD increasing 17,000-fold between that and the most significantly associated SNP (Supplemental Table 2; *BovineHD2000010982* and *BovineHD2000010897*) within slick individuals. Another potential candidate gene is the *sperm flagellar 2*, or *SPEF2* gene. While the *SPEF2* gene is predominantly associated with spermatogenesis defects in mice and pigs (Sironen et al., 2010, 2012) it was also implicated as a partially duplicated gene in late feathering male chickens (Elferink et al., 2008). Relatively little is known about the protein but it has been suggested that the ATP/GTP binding site and proline rich domain may be involved in signal transmission as well (Ostrowski et al., 1999). In regards to our analyses, the most highly associated SNP, *BovineHD2000010982* (Supplemental Table 1), which was also part of the highly associated haplotype Block 120 (Table 2), were both within *SPEF2*. As mentioned before with *SKP2*, this SNP exponentially increased in LD to SNP *BovineHD2000010897* in SKP2 with respect to slick phenotyped animals.

In total, this study used five different approaches for defining and validating the *SLICK* locus and generated a consensus region of less than 1 Mb. The study validated previous findings in regards to the general *SLICK* region and Senepol signatures of selection and ancestry but used an assortment of analyses and new tactics to narrow the *SLICK* locus. The causative mutation remains elusive with associated SNPs found in this study being mostly located in non-coding regions. The disparities in population structure and shifting of the GWAS peak and IBS regions when comparing Senepol and Romosinuano versus the Carora raise issues as to when and how many times SLICK phenotype was selected. Our results suggest the possibility of two separte post-breed mutations versus a single pre-breed mutation formation. Bovine polledness recently exemplified this theory with two distinct genetic haplotypes aligning with either Celtic or Fresian breed formation (Medugorac et al., 2012). It also raises the possibility of potentially more than one mutation being responsible for the slick hair phenotype. Due to the increase in statistical association with the inclusion of Carora in the analysis, we hypothesize that the same gene is controlling the slick phenotype in all breeds but one mutation is responsible for the trait in the Senepol and Romosinuano while a second mutation may be responsible for the trait in Carora. Fine mapping of the *SKP2* and *SPEF2* genes or targeted sequencing of the established *SLICK* locus is necessary to identify potential causative mutations such as SNPs and/or indels either altering the protein product itself or regulating gene function. Gene expression studies would be an appropriate follow-up for causative gene validation based on fine-mapping results. The inclusion of Criollo Limonero cattle, which is being pursued at this time, would benefit both the investigation of the ancestral origins of the slick phenotype and give a “pure,” non-composite, slick-haired criollo lineage to compare and identify causative mutations. Recent insight pertaining to breed purity of Carora herds and phenotype differences with respect to seasonal hair shedding confounded phenotyping and should be taken into consideration for future work. Industry gains include potential SNPs and haplotype blocks as new diagnostic markers for identifying the state of inheritance of SLICK for guided breed management. Identifying *SKP2* as a candidate gene lends new insight into the potential mechanisms for keratinocyte development or *SPEF2* involvement in signal transmission and their potential influence on thermo-regulation in tropical cattle.

## Author contributions

The authors have made the following declarations about their contributions: Conceived and designed the experiments: Heather J. Huson, Eui-Soo Kim, Robert W. Godfrey, Timothy A. Olson, Chad C. Chase, Tad S. Sonstegard. Performed experiments: Heather J. Huson, Matthew C. McClure, Tad S. Sonstegard. Analyzed the data: Heather J. Huson, Eui-Soo Kim, Tad S. Sonstegard. Data acquisition and interpretation: Robert W. Godfrey, Timothy A. Olson, Chad C. Chase, Rita Rizzi, Ana M. P. O'Brien, Curt P. Van Tassell, José F. Garcia, Tad S. Sonstegard. Wrote the paper: Heather J. Huson, Eui-Soo Kim, Tad S. Sonstegard.

### Conflict of interest statement

The authors declare that the research was conducted in the absence of any commercial or financial relationships that could be construed as a potential conflict of interest.
