# Supplementary material for: Genome-wide association study and ancestral origins of the slick-hair coat in tropically adapted cattle
Source: Front Genet. 2014 Apr 29;5:101. doi: 10.3389/fgene.2014.00101 (PMC4010767; doi:10.3389/fgene.2014.00101)
Supplement: Supplementary file 1 [file DataSheet1.PDF]

Genome-wide association study and ancestral origins of the slick-hair coat in tropically adapted cattle

Supplementary Material

Supplemental Figure 1. Unsupervised clustering analysis of Senepol and their ancestral breeds.

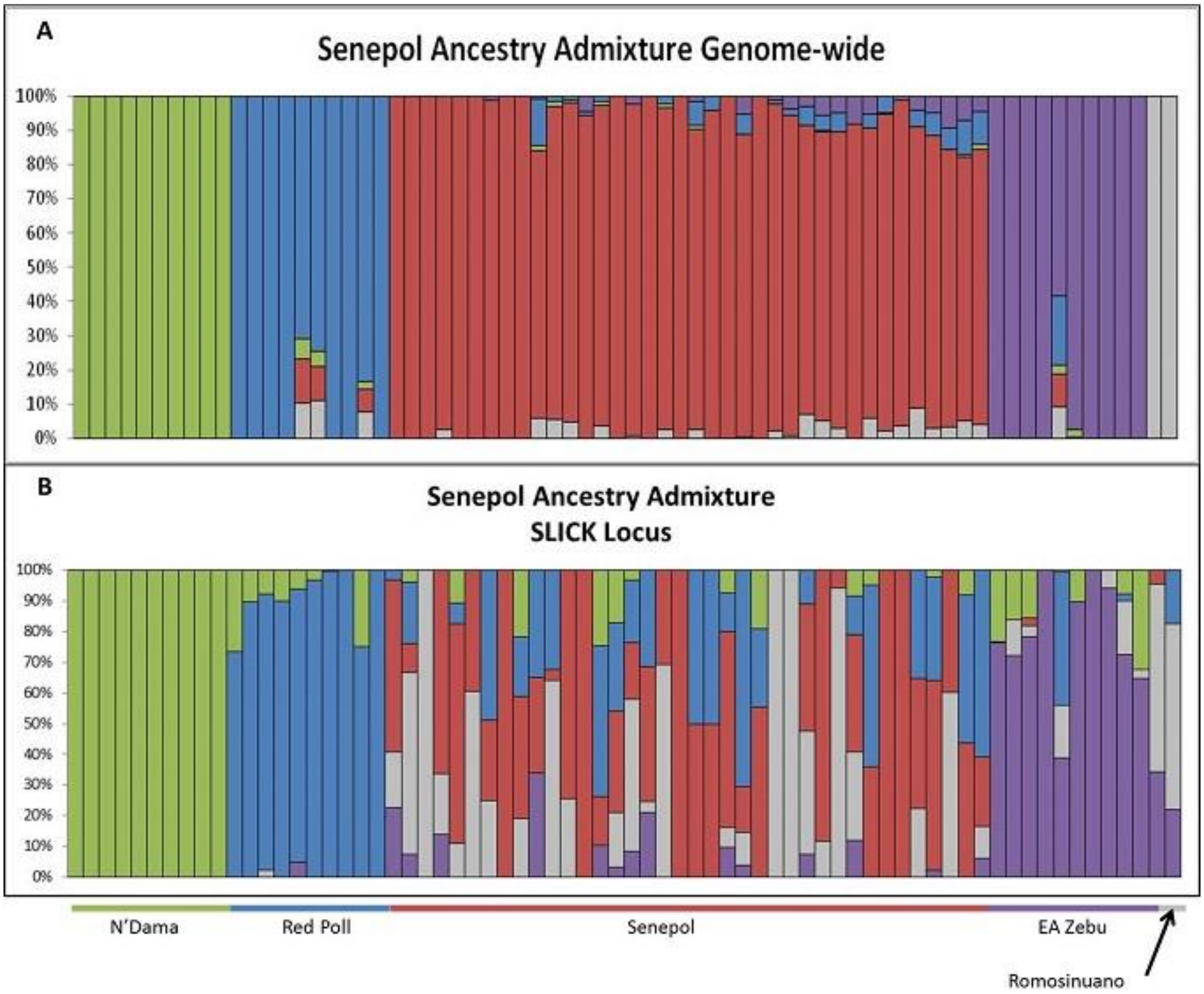

A.) A genome-wide admixture analysis of 10-N'Dama, 10-Red Poll, 38-Senepol, 10-East African Zebu, and 2-Romosinuano utilizing 639,663 SNPs. Each color represents a distinct genetic population; N'Dama-green, Red Poll-blue, Senepol-red, EA Zebu-purple, Romosinuano-grey. B.) A comparative admixture analysis of the same individuals utilizing only 4,351 SNPs spanning *SLICK* on Chr20. Individual animal admixture increased particularly in the Senepol, EA Zebu, and Romosinuano with lower population values and generally less defined populations with the exception of the N'Dama and Red Poll.

Supplemental Figure 2. Principle component analysis (PCA) of slick and non-slick animals used in the genome-wide association study.

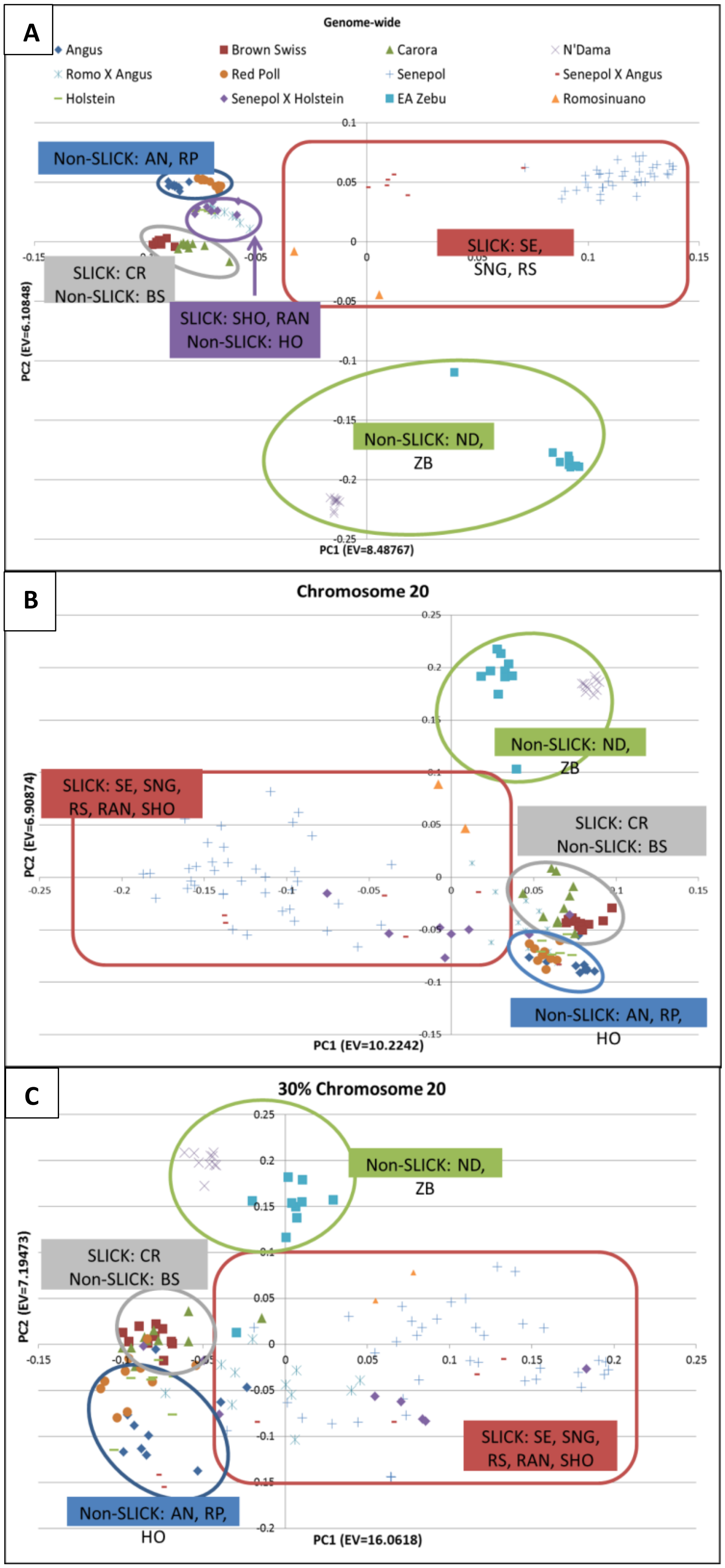

Population structure was examined using PCA to identify variation in relatedness among slick and non-slick animals. Marks denote individual animals with color and shape representing the different breeds. X-axis is principle component 1 and y-axis is principle component 2. Boxes and circles highlight breed clusters and slick phenotype within the cluster. A.) PCA using 639,663 SNPs dispersed genome-wide. B.) PCA using 19,475 SNPs dispersed across Chr 20. C.) PCA using 4,351 SNPs dispersed across an 18 Mb region targeting *SLICK*.

Supplemental Figure 3. Consensus identity by state (IBS) haplotypes between Senepol and Carora at SLICK.

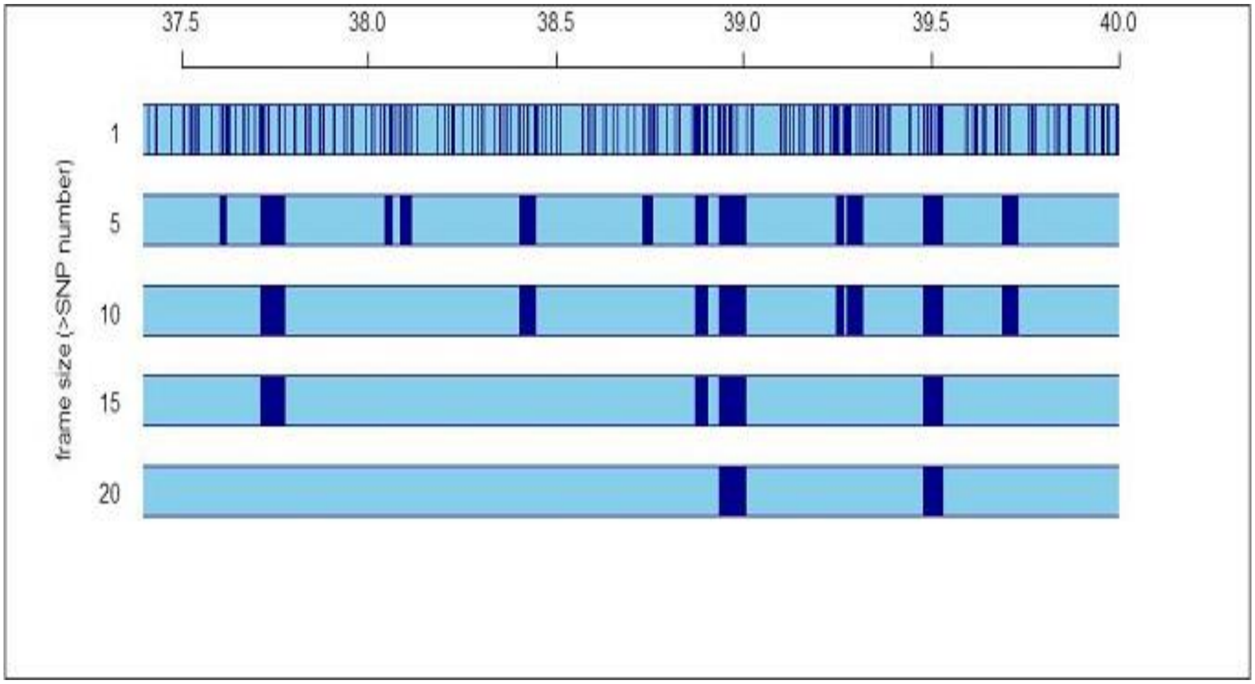

Common IBS regions (dark blue) were identified in Senepol and Carora using an increasing SNP window of 1, 5, 10, 15, and 20 SNPs. Only two blocks were apparent at the 20 SNP threshold and no common haplotype blocks were found with greater than 25 SNPs.

Supplemental Table 1. The 35 most highly associated SNPs from a genome-wide association study of the slick phenotype. SNP ID and location are identified with associated minor allele and respective frequency in either slick-haired or non-slick-haired study populations. Statistical association is denoted with p-values and respective $-\log^{10}$p-values used for the Manhattan plot.

Chromosome	Base Pair	SNP ID	Minor Allele	MAF SLICK	MAF Non-SLICK	p-value	"-log10p-value"
20	34928482	Hapmap47027-BTA-50369	G	0.8889	0.3525	3.00E-08	7.523171
20	37179958	BovineHD2000010654	A	0.625	0.08197	6.16E-07	6.210586
20	37226278	BovineHD2000010663	G	0.5278	0.008197	7.72E-07	6.112479
20	37249668	BovineHD2000010665	G	0.5208	0.02459	6.16E-07	6.210586
20	37313868	BovineHD2000010676	C	0.5278	0.03279	6.16E-07	6.210586
20	37342386	BovineHD2000010680	G	0.5278	0.03279	6.16E-07	6.210586
20	37357181	BovineHD2000010683	C	0.5278	0.04918	6.16E-07	6.210586
20	37382010	BovineHD2000021323	A	0.5486	0.1148	6.16E-07	6.210586
20	37858644	BovineHD2000010832	A	0.06944	0.4836	1.47E-08	7.833412
20	38098322	BovineHD2000010894	A	0.06944	0.4672	5.66E-07	6.247057
20	38112689	BovineHD2000010897	A	0.05556	0.4016	2.68E-11	10.572275
20	38243418	BovineHD2000010917	A	0.8472	0.3689	8.87E-10	9.052200
20	38421175	BovineHD2000010958	A	0.8403	0.3607	9.64E-08	7.016131
20	38425311	BovineHD2000010960	G	0.8472	0.3689	1.18E-07	6.928987
20	38440030	BovineHD2000010962	A	0.6458	0.1557	1.07E-07	6.969466
20	38544165	BovineHD2000010982	G	0.5208	0.05738	1.54E-13	12.811880
20	39262981	BovineHD2000011163	C	0.5208	0.05738	2.64E-07	6.577841
20	39263700	BovineHD2000011164	A	0.5208	0.05738	6.36E-07	6.196467
20	39264432	BovineHD2000011165	G	0.5208	0.05738	6.36E-07	6.196467
20	39267121	BovineHD2000011167	G	0.5208	0.05738	5.04E-07	6.297698
20	39270125	BovineHD2000011170	G	0.5208	0.05738	8.33E-07	6.079332
20	39271011	BovineHD2000011171	G	0.1528	0.623	8.33E-07	6.079332
20	39272048	BovineHD2000011172	G	0.1528	0.623	8.33E-07	6.079332
20	39272791	BovineHD4100014689	A	0.5208	0.06557	8.33E-07	6.079332
20	39273517	BovineHD2000011173	A	0.8403	0.3689	8.33E-07	6.079332
20	39275917	ARS-BFGL-NGS-65409	G	0.8403	0.3689	8.33E-07	6.079332
20	39279829	BovineHD2000011177	A	0.8403	0.3689	3.48E-07	6.458522
20	39280821	BovineHD2000011178	A	0.8403	0.3689	8.33E-07	6.079332
20	39282257	BovineHD2000011179	A	0.8403	0.3689	8.33E-07	6.079332
20	39283526	BovineHD4100014690	G	0.8403	0.3689	8.33E-07	6.079332
20	39284199	BovineHD2000011180	C	0.8403	0.3689	8.33E-07	6.079332
20	39308172	BovineHD2000011184	G	0.8403	0.3689	9.41E-07	6.026548
20	39337264	BovineHD2000021078	A	0.8403	0.3689	2.96E-08	7.528284
20	39341194	BovineHD2000011191	G	0.8403	0.3689	1.67E-08	7.777502
20	39345284	BovineHD2000011192	A	0.1528	0.623	1.67E-08	7.777502

Supplemental Table 2. Linkage-disequilibrium (LD), r^2, between the top associated SNPs from the *SLICK* genome-wide association study. A) Pairwise r2 values within the non-slick-haired study population. B) Pairwise r2 values within the slick-haired study population. C) The magnitude of increased pair-wise LD in a comparison of slick versus non-slick individuals.

**A.**	**non-SLICK**		
**R^2**	**BovineHD2000010897**	**BovineHD2000010917**	**BovineHD2000010982**
**BovineHD2000010897**		**0.092450**	**0.000009**
**BovineHD2000010917**			**0.014605**
**BovineHD2000010982**			
**B.**	**SLICK**		
**R^2**	**BovineHD2000010897**	**BovineHD2000010917**	**BovineHD2000010982**
**BovineHD2000010897**		**0.665282**	**0.156563**
**BovineHD2000010917**			**0.145956**
**BovineHD2000010982**			
**C.**	**Magnitude of Increased LD between SLICK and non-SLICK**		
	**BovineHD2000010897**	**BovineHD2000010917**	**BovineHD2000010982**
**BovineHD2000010897**		**7**	**17,313**
**BovineHD2000010917**			**10**
**BovineHD2000010982**			
